# Discussion about different cut-off values of conventional hamstring-to-quadriceps ratio used in hamstring injury prediction among professional male football players

**DOI:** 10.1371/journal.pone.0188974

**Published:** 2017-12-07

**Authors:** Monika Grygorowicz, Martyna Michałowska, Tomasz Walczak, Adam Owen, Jakub Krzysztof Grabski, Andrzej Pyda, Tomasz Piontek, Tomasz Kotwicki

**Affiliations:** 1 Department of Spondyloortopaedics and Biomechanics of the Spine, Poznan University of Medical Sciences, Poznań, Poland; 2 Rehasport Clinic, FIFA Medical Centre of Excellence, Poznań, Poland; 3 Institute of Applied Mechanics, Poznan University of Technology, Poznań, Poland; 4 Spine Disorders and Paediatric Orthopaedics Department, Poznan University of Medical Science, Poznań, Poland; Universidad Europea de Madrid, SPAIN

## Abstract

**Objective:**

To measure the sensitivity and specificity of differences cut-off values for isokinetic H_con_/Q_con_ ratio in order to improve the capacity to evaluate (retrospectively) the injury of hamstring muscles in professional soccer screened with knee isokinetic tests.

**Design:**

Retrospective study.

**Methods:**

Medical and biomechanical data of professional football players playing for the same team for at least one season between 2010 and 2016 were analysed. Hamstring strain injury cases and the reports generated via isokinetic testing were investigated. Isokinetic concentric(con) hamstring(H) and quadriceps(Q) absolute strength in addition with H_con_/Q_con_ ratio were examined for the injured versus uninjured limbs among injured players, and for the injured and non-injured players. 2 x 2 contingency table was used for comparing variables: predicted injured or predicted uninjured with actual injured or actual uninjured. Sensitivity, specificity, accuracy, positive and negative predictive values, and positive and negative likelihood ratio were calculated for three different cut-off values (0.47 vs. 0.6 vs. 0.658) to compare the discriminative power of an isokinetic test, whilst examining the key value of H_con_/Q_con_ ratio which may indicate the highest level of ability to predispose a player to injury. McNemar’s chi^2^ test with Yates’s correction was used to determine agreement between the tests. PQStat software was used for all statistical analysis, and an alpha level of p <0.05 was used for all statistical comparisons.

**Results:**

340 isokinetic test reports on both limbs of 66 professional soccer players were analysed. Eleven players suffered hamstring injuries during the analysed period. None of these players sustained recurrence of hamstring injury. One player sustained hamstring strain injury on both legs, thus the total number of injuries was 12. Application of different cut-off values for H_con_/Q_con_ significantly affected the sensitivity and specificity of isokinetic test used as a tool for muscle injury detection. The use of 0.47 of H_con_/Q_con_ as a discriminate value resulted in significantly lower sensitivity when compared to 0.658 threshold (sensitivity of 16.7% vs. 91.7%, respectively; t = 6.125,p = 0.0133). Calculated values of specificity (when three different cut-off were applied) were also significantly different. Threshold of 0.6 of H_con_/Q_con_ resulted with significantly lower specificity compared to 0.47 value (specificity of 46.9% vs. 94.5%, respectively; t = 153.0,p<0.0001), and significantly higher specificity when compared to 0.658 (specificity of 46.9% vs. 24.1%, respectively; t = 229.0, p<0.0001).

**Conclusion:**

The use of different cut-off values for H_con_/Q_con_ significantly affected the sensitivity and specificity of isokinetic testing. The interpretation of usefulness of isokinetic test as a screening tool in a group of male professional football players to predict hamstring injury occurrence within the next 12 months might be therefore significantly biased due to the different threshold values of H_con_/Q_con_. Using one “normative” value as a cut-off (e.g. 0.47 or 0.60, or 0.658) to quantify soccer players (or not) to the group with a higher risk of knee injury might result in biased outcomes due to the natural strength asymmetry that is observed within the group of soccer players.

## Introduction

Muscle strain or ligament rupture are known to be extremely common injuries in elite professional soccer [1]. These injury types are generally caused by intense high-speed actions, quick force production with multiple directional changes during accelerating, decelerating, jumping or kicking [2,3]. These specific athletic movements induce stress and require the prime mover hamstrings to work in position of extreme stretching [4]. Within recent literature isokinetic evaluation is one of the most discussed methods used to assess the risk of muscle injury with hamstring-to-quadriceps (H/Q) ratio being the key muscle injury indicator collected during the isokinetic test [5–9].

Prospective studies conducted by Fousekis et al. and Croisier et al. on a cohort of 100 players from 4 teams of Greek 3^rd^ Division^**6**^ and on 462 players from Brazilian, Belgian and French leagues [7], respectively, support the usefulness of isokinetic testing in estimating the risk of muscle injury. Dauty et al. [9], in another case-control study where 350 isokinetic tests were performed in 136 footballers at the beginning of soccer season, confirmed that isokinetic tests are useful for predicting the likelihood of hamstring injury in professional soccer players during the competitive season. Ardern et al. confirmed that H/Q strength imbalance may impact in-season football performance and could have implications for the future risk of injury [10].

Contrarily, in two recent important papers published by Freckleton and Pizzari [11] and van Dyck et al. [12] H/Q ratio was not identified as a risk factor for hamstring injuries. Freckleton and Pizzari completed meta-analysis using five studies, and they demonstrated no difference between groups with decreased H/Q ratio vs. increased H/Q ratio and hamstring injury [11]. A 4-year cohort study performed by van Dyk et al. on a large sample of total 614 professional soccer players does not support the use of isokinetic testing to determine the relation between strength difference and hamstring injuries [12]. All the above shows that different methods used by researchers to assess the risk of injury might influence the consistency of the studies and might lead to contradictory results. Particularly, various values of outcomes considered as injury indicators are differently classified by authors in determining the cut-off between the players with an increased risk of injury and those who are not at risk; this might influence results and conclusions in the available studies.

Additionally, to verify the usefulness of isokinetic test as a potential tool for assessing muscle injury risk, we have to discuss broadly the cut-off values of H/Q ratio as the discriminative parameter. Some researchers calculate the area under the curve (AUC) of receiver operating characteristics (ROC) based on their raw data, to examine the discriminative power of an isokinetic test and to find the value of H/Q ratio that may predispose a player to injury [12,13]. Other authors use previously set cut-off points [8,9] to verify if a player is at risk of muscle injury, and additionally calculate sensitivity and specificity of isokinetic tests in muscle injury prediction [14].

Usually, in soccer specific studies, two thresholds of H/Q ratio are used as the normative cut-off values: 0.60 and 0.47 for knee concentric flexion-extension movements at 60deg/s of isokinetic velocity. It was Klein and Allman in 1969 who first proposed a concentric hamstring-to-quadriceps ratio of 0.60, as reported by several authors [15–17]. They suggested that the proportion of muscle strength of knee flexors and knee extensors should be equal to or greater than 60% in order to prevent possible muscle strain injuries [15]. Dauty et al. [8,18] used a fixed threshold of H_con_/Q_con_ ratio set at 0.6 to analyse whether soccer players were at risk of hamstring injury or not. They decided to use the 0.6 cut-off value, as it was previously used in the studies of Heiser et al. [15], and Orchard et al. [14], and Bennel et al. [19]. However, it is questionable whether the results of these studies can be applied in further publications regarding the prediction of soccer-related muscle injury based on isokinetic test, as all three studies [14,15,19] were performed on non-soccer populations. Additionally, there are other limitations concerning the study design and result interpretation. Heiser et al. used the value of H_con_/Q_con_ ratio of 0.6 as the normative value of knee unilateral ratio [15]. However, the aim of this study was to review the number of hamstring injuries prior to and after the use of isokinetic assessment for muscle imbalance: the athletes who did not meet the normative criteria were put through isokinetic rehabilitation program designed to correct the imbalance. Moreover, no comparison of H_con_/Q_con_ values in players with and without hamstring strains were provided, thus the prediction of muscle injury based on isokinetic results from this study might be biased. Orchard et al. suggested that players had a substantially increased risk of hamstring injury when their H_con_/Q_con_ was lower than 0.61 in the preseason concentric isokinetic test [14]. However, deeper analysis of mean values and standard deviation for injured and uninjured legs (0.550±0.065 vs. 0.662±0.071, respectively) shows different information and makes these “normative” values more difficult to apply in further studies.

The other, commonly used cut-off value of conventional H_con_/Q_con_ ratio– 0.47 –was set by Croisier et al. [20] and then it was applied in different studies [7,9,10,21–23]. However, it is important to be aware of the origins and adaptation of this cut-off value. Tracing its history backwards we learn that Dauty et al. [9]decided to use conventional ratio fixed at 0.47 on 136 footballers in reference to the study of Croisier et al. [7] published in 2008. Similarly, Ardern et al. [10] applied the same fixed ratio to define hamstring strength imbalance in 42 professional male soccer players from Australian national level club. In 2008 Croisier et al. performed a prospective study on a large sample of 687 professional soccer players, and they used the 0.47 H_con_/Q_con_ value based on the criteria described by the Croisier et al. in 2002 [21]. However, in 2002 Croisier et al. observed only 26 athletes with a history of hamstring muscle injury and recurrent strains and discomfort [21], and only 14 of them were soccer players. In this study, muscle imbalance potentially leading to hamstring injury was determined using the similar 0.47 cut-off value based on study of Croisier et al. published in 1999. In 1999 Croisier et al. performed a reliability study on the group of sedentary people and physical education students [20]. The subjects realized two successive evaluations (one week apart) in concentric mode and ten days after in eccentric mode. Excellent reproducibility during concentric contractions of the quadriceps and the flexors was found. Unfortunately, there was no follow-up registration of muscle injury applied in this study [20]. Summing up, it seems questionable to use “the normative” value of H_con_/Q_con_ which was adapted from non-footballers with no muscle strain follow up to verify the risk of hamstring muscle injury in professional soccer players based on isokinetic assessment. Moreover, different authors report higher values of H_con_/Q_con_ in football players (Table 1), and thus, all the results from studies using the value of 0.47 H_con_/Q_con_ might be biased.

**Table 1:** Values of concentric H/Q ratio used in professional soccer players with and without hamstring injury (mean±SD) at 60 deg/s of isokinetic velocity during concentric knee flexion–extension movement.

Authors	Mean age of players (range) [years]	Injured		Non-injured		Type of study	Comments
		n	Mean ± SD (range)	n	Mean ± SD (range)		
Paton et al. 1989 [24]	19.9	14	0.7 (0.5–1.0)	29	0.7 (0.5–1.0)	Cross-sectional	With retrospective injury register, number of injured or uninjured legs
Mangine et al. 1990 [25]				31	0.56±0.17(R)	Cross-sectional	Cross-sectional assessment repeated for 5 years, leg right(R), left(L)
					0.56±0.17(L)		
Zakas et al. 1995 [26]	21.0–26.6			51	0.68±0.09(I)	Cross-sectional	Four different divisions (I,II,III,IV)
					0.71±0.09(II)		
					0.72±0.10(III)		
					0.67±0.06 (IV)		
Tourny-Chollet et al. 2000 [27]	22			21	0.64(NP)	Cross-sectional	H/Q values calculated indirectly, preferred(P), non-preferred(NP) leg
					0.66(P)		
Dauty et al. 2003 [8]	23	11	62.2 ± 12.5	17	66.8±9.0	Prospective	Number of injured and uninjured players
Dauty et al. 2003 [18]	23	15		17	0.67±0.07 (UI)	Prospective	Uninjured legs (U) of injured (I) and uninjured (UN) players
					0.66±0.09 (UUN)		
Lehance et al. 2008 [23]	26			19	0.62±0.07(P) vs.	Cross-sectional	Three groups of professional players: PRO, U-21 and U-17; preferred(P) vs. non-preferred(NP) leg
					0.59±0.07 (NP) (PRO)		
	19.5			20	0.60±0.07(P) vs.		
					0.61±0.08 (NP) (U-21)		
	15.7			18	0.63±0.07(P) vs		
					0.61±0.08 (NP) (U-17)		
Fousekis et al. 2011 [6]	24			100	0.56±0.80(YR)	Cross-sectional	Number of injured and uninjured players; H/Q values are provided depending on professional years of playing: young(Y)5-7 yrs, medium(M)8-10yrs, old(O)more or equal to 11 yrs, and testd leg: right(R) vs. left(L)
					0.55±0.10(YL)		
					0.56±0.80(M)		
					0.58±0.70(M)		
					0.58±0.80(OR)		
					0.59±0.10(OL)		
Henderson et al. 2010 [28]	23	10	0.60±0.09	25	0.62±0.12	Prospective	All results are presented for preferred leg(P)
da Fonseca et al. 2007 [29]	24			117	0.83±0.19(P)	Cross-sectional	preferred(P), non-preferred(NP) leg
					0.51 ± 0.09(NP)		
Zabka et al. 2011 [30]	24			39	57.8±0.08(R)	Cross-sectional	right(R) vs. left(L), number of uninjured players
					57.7±0.07(L)		
Ruas et al. 2015 [31]	26			102	0.60±0.07(GP)	Cross-sectional	Depending on players position: goalkeepers(G), side backs(SB), central backs(CB), central defender midfielders(CDM), central attacking midfielders(CAM), forwards(F) and tested leg preffered(P) vs. non-preffered(N)
					0.55±0.08(GN)		
					0.63±0.16(SBP)		
					0.61±0.10(SBN)		
					0.64±0.13(CBP)		
					0.61±0.12(CBN)		
					0.60±0.13(CDMP)		
					0.62±0.09(CDMN)		
					0.62±0.12(CAMP)		
					0.60±0.08(CAMN)		
					0.59±0.11(FP)		
					0.58±0.12(FN)		
Carvalho et al. 2016 [4]	25.5			159	0.62 ± 0.10 (IR)	Cross-sectional	Depending on the level of league (I vs. II) and tested leg right(R) vs. left(L)
					0.61 ± 0.11 (IL)		
					0.59 ± 0.10 (IIR)		
					0.58 ± 0.09 (IIL)		
Dauty et al. 2016 [9]	22.5(U); 25.2(I)	64	0.66 ± 0.11	620	0.66 ± 0.10	Case-control	Number of injured and uninjured legs

Lately, McCall et al. classified isokinetic muscle testing as an insufficient evidence to assign a specific recommendation for its use in the practical setting [5], especially in muscle injury prediction. Additionally, there is increasing evidence that using isokinetic tests in preseason screening might not be sufficient to predict muscle injury [11,12]. However based on the information provided in the paragraphs above, the question arises whether these recommendations were based on appropriately interpreted data. Perhaps a certain degree of misinterpretation might be observed due to the improper cut-off points used by the cited authors. Taking into consideration all the above, we developed the following research question: is there a possibility that hamstring injury risk analysis in male professional soccer players screened with knee isokinetic tests might be wrong due to incorrectly used normative values? That is why we aimed to measure the sensitivity and specificity of differences cut-off values for isokinetic H_con_/Q_con_ ratio in order to improve the capacity to evaluate (retrospectively) the injury of hamstring muscles in professional soccer. We hypothesised that using different cut-off values for H_con_/Q_con_ will significantly affect the sensitivity and specificity of isokinetic test used as a tool for muscle injury detection.

## Material and methods

### Participants

The team included in this study competed in the Polish Premier League (Ekstraklasa), the highest level of the male professional soccer competition in Poland. The team has been systematically evaluated at the beginning of each part of the soccer season for consecutive seven seasons. In first four seasons (2010–2013) the team played 30 matches in the league. In past three seasons (2014–2016) the team had played 37 regular matches in the national league, since the Polish Football Association redesign the form of the elite league. The team achieved similar rounds in national cups, usually achieved at least quarterfinal-round and played from one to seven more matches during the analysed seasons. Additionally, the team competed in the European leagues (typically achieved 2^nd^ qualification round of UEFA Champion League or UEFA Europa League), usually played four extra matches in the whole season (once–no participation, three seasons–four matches, one season–six, one season 12 matches, and once–fourteen additional matches). During the analysed period the team played on average 43 matches per season (from 34 to 52 matches). The number and the intensity of matches played in different types of competition were thus different throughout these seven seasons. During the season the players trained for 10–14 hours weekly, and they played one or two games weekly. All players followed the same scheme of the training. During the warm-up and cool-down the players performed some injury prevention strategies, such as neuromuscular training or stretching. The strength and conditioning coach usually supervised these exercises during the training or match. The tactics exercises were performed in position-specific groups, managing by the main coach. The training session usually lasted for 90–120 minutes. All players were screened for biomechanical and functional indicators of neuro-muscular level of performance. Individual playing positions and anthropometric data were collected. The routine biomechanical evaluation consisted of functional movement screen tests, knee hamstring and quadriceps isokinetic strength and endurance tests, proprioception tests and ground reaction force analysis. This evaluation was usually performed twice a year, during the preseason period from January to February, and from June to July, with the official start of the season in August or September each year.

### Study design

This single-centre study was performed using retrospectively collected clinical data on professional football players. Medical and biomechanical data of athletes, members of one professional football team, who played for this team for at least one season between 2010 and 2016 were analysed. Between March 2016 and May 2016 we have analysed football players’ injury/medical history focusing on hamstring strain, which is confirmed as one of the most typical for elite male football population [32]. We have also investigated athlete’s comprehensive reports obtained from biomechanical tests previously performed on isokinetic dynamometer.

### Ethical approval

Polish ethical guidelines do not provide defined regulations for observational studies including the analysis of records containing biomedical or other information. There is no obligation to receive positive decisions of Bioethical Committee, however, we decided to notify the Bioethical Committee that we were going to perform a study involving retrospective analysis of biomedical information. The Bioethical Committee analysed the principles and study design of the scientific project, and we received a letter of confirmation that the study did not meet the criteria for an experimental study, and thus it could be conducted without a decision from the Bioethical Committee (S1 File).

### Isokinetic tests

Isokinetic tests were conducted either as a part of the baseline testing for club transfer decisions, or as a part of the routine biomechanical prospective evaluation. All tests were performed on a Biodex System 3 Pro isokinetic dynamometer (Biodex Corp, 49 Natcon Drive, P.O. Drawer S, Shirley, NY). The warm-up that each player carried out before the isokinetic assessment took 10–15 minutes and consisted of mild pedalling on a stationary Monark cycle ergometer at a moderate pace (50–100 W) and dynamic stretches for the major lower limb muscle groups. Warm up and preparation phase was the same as previously described [33]. All testing procedures concerning patient’s position, alignment axis of dynamometer rotation, stabilization, and gravity correction were conducted following the guidelines described in the literature [34]. Three repetitions of knee concentric flexion and concentric extension movement were performed through a knee range of motion (ROM) of 0° (flexed) to 90° (full extension) at the 60° s-1 angular velocity. All players performed three trials at sub-maximal efforts with a gradually increasing load (50%, 75%, and approximately 100% of maximum capability) and then they performed one set of the knee extension-flexion movements at the maximal level. All participants started to follow the test protocol using randomly chosen leg, and then it was changed to the opposite side. Players were also verbally encouraged to complete the movement in full ROM. Subsequently, the same protocol was followed with the opposite leg. A 30-s rest was given after the third sub-maximal trial and a 3-min break was given when the machine setting was changed for the opposite leg. All tests were conducted in the same order for each player, and all tests took place before 12 am to exclude inter-day variability [35]. Different examiners, all regular physiotherapists in the clinic where football players were routinely tested, performed the testing. Examiners graduated from specialized, additional biomechanical evaluation courses and each of them had at least 3 years of experience in isokinetic testing of the knee and other joints. To reduce potential inter-examiner induced variability, standardized instructions were given by examiners to the football players. The players were not subjected to a higher training load for one day before the measurements. Values of hamstring-to-quadriceps conventional ratios (H_con_/Q_con_) were selected for further analysis, as this outcome is suggested to be one of knee injury risk determinants [5–9].

### Types of injuries

One medical centre provided medical services for all players from this team, because the clinic is the medical partner for the football club, so it was possible to standardize injury diagnosis and treatment. This made the medical records suitable for this research. We searched the medical documentation and looked for hamstring strain injury cases. Sports medicine practitioner and/or team physicians recorded hamstring strain injury based on the clinical examination (identifying pain on palpation, pain with isometric contraction, and pain with muscle lengthening), and all injuries were confirmed by ultrasound examination. The USG examination was performed by a sports radiology specialist using USG HD11 XE Philips device (Bothwell, USA) with 12–5 MHz broadband linear array ultrasound scan head. A player was classified as injured if he was unable to take part in a match or in training session due to hamstring strain that happened in a football match or during training, and at least one of the following consequences had been present: decrease in the quantity or level of sports activity for at least one day, or need for medical evaluation or non-operative or operative treatment [36]. All hamstring injuries sustained by every football player within the 12-month period after the isokinetic evaluation were counted.

### Statistical analysis

Hamstring and quadriceps absolute strength values for each player were collected from comprehensive reports generated by Biodex software. We calculated mean and standard deviation (SD) of the ratios for the hamstring injury and non-hamstring injury groups. Injured limbs were also compared with uninjured limbs among injured players. Absolute values (peak torque) were also adjusted for body weight as previously described [12]. We performed the calculation for three different cut-off values: two of them: 0.47 and 0.6 were commonly used in injury risk analysis thorough isokinetic assessment, and were used in previously published reports dedicated to injury prediction in football players [7–10,18,21]. The third value of cut-off was established according to the methodology described in the next paragraph.

Receiver operating characteristic (ROC) curve analysis was performed to define the optimal cut-off value for H_con_/Q_con_ ratio obtained from isokinetic tests completed by our study group. We used two criteria to determine the optimal cut-off value objectively, as it is recommended. The first criterion was ‘the closest to (0, 1) criterion’, which represents the values at the shortest distance from the upper left corner to the ROC curve (UL index). The second criterion was ‘the Youden index’, which describes the maximum vertical distance between the ROC curve and the diagonal or chance line [37].

Discriminant-function analysis was performed using the variables of lower H_con_/Q_con_ ratio at 60 deg/sec of isokinetic test to classify limbs into potentially injured versus potentially noninjured. We have used the 2 x 2 contingency table for comparing variables: predicted injured (when H_con_/Q_con_ values were lower than the cut-off threshold) or predicted uninjured (when H_con_/Q_con_ values were higher or equal to the cut-off threshold) with actual injured (when hamstring strain injury was sustained within 12 month after isokinetic evaluation) or actual uninjured (when hamstring strain injury was not sustained within 12 month after isokinetic evaluation). We calculated sensitivity, specificity, accuracy, positive and negative predictive values, and positive and negative likelihood ratio for all three cut-off values to compare the discriminative power of an isokinetic test, and to find the value of H_con_/Q_con_ ratio that indicate the highest level of ability to predispose a player to injury. McNemar’s chi^2^ test with Yates’s correction was used to determine agreement between the tests. It was performed separately for injured and non-injured data to compare the sensitivities and specificities between different cut-offs values. Quantitative measure of the strength of an observed occurrence was calculated by effect size and was interpreted as small (0.2–0.3), medium (0.5), or large (>0.8) [38]. We used PQStat software for all statistical analysis, and an alpha level of p <0.05 was used for all statistical comparisons.

## Results

### Participants

Medical and biomechanical data of 74 professional male football players (aged 23.42±4.63 years old, height 182.91±5.40 cm, weight 77.80±6.60 kg) were analysed in this study. The medical documentation of 2 players was missing from the archive, the period of observation of 6 players was too short (less than 12 months) due to a transfer to another team or career termination, so records of 8 players were rejected. In total we collected 340 isokinetic test procedures on both limbs of 66 professional football players. In this cohort, 87.88% of players were of European origin (n = 58), with minor ethnic representations from Africa (9.09%, n = 6), Central America (1.52%, n = 1), and South America (1.52%, n = 1). Playing position was documented in 4 categories: goalkeepers (13.63%, n = 9), defenders (28.78%, n = 19), midfielders (37.87%, n = 25), and forwards (19.69%, n = 13). 21.21% of players were left dominant (n = 14), 71.21% were right dominant (n = 47), and 7.58% declared no lower limb domination (n = 5).

### Isokinetic strength measurements

Absolute and relative adjusted for body weight (BW) isokinetic muscle strength values for injured and non-injured players are presented in Table 2.

**Table 2:** Values of absolute values and relative–adjusted for body weight (BW)–isokinetic parameters in injured and non-injured players.

	Injured players (n = 11)				Uninjured players (n = 55)		
	Injured Limb	Uninjured Limb	Absolute Difference	Effect size (d)	Both limbs	Absolute Difference	Effect size (d)*
**Quadriceps**							
**Concentric**^**a**^ **at 60 deg/s**	261.89±13.01	275.72±25.63	13.83	0.68	247.32±37.77	28.4	0.87
**BW adjusted**	344.39±37.80	359.79±18.94	15.4	1.12	313.74±44.74	46.05	1.34
**Hamstring**							
**Concentric at 60 deg/s**	151.58±19.00	152.54±24.16	0.96	0.05	147.69±23.00	4.85	0.20
**BW adjusted**	197.87±20.18	198.51±23.33	0.64	0.02	187.51±28.71	11.00	0.42
**H**_**con**_**/Q**_**con**_ **ratio**							
**Absolute values**	0.58±0.09	0.55±0.06	0.03	0.39	0.6±0.1	0.05	1.26

aAbsolute values and values adjusted for body weight (BW) are shown in newton-meters as mean±SD.

*effect size calculated compared to uninjured limb

### Hamstring strain injuries

The database included information about hamstring injuries, sustained or not within 12 months after the isokinetic examination. Eleven players (16.67%) suffered hamstring injuries during the analysed period. None of these players sustained recurrence of hamstring injury. One player sustained hamstring strain injury on both legs, thus the total number of injuries was 12.

### Cut-off value

ROC analysis performed on our study group revealed an H_con_/Q_con_ value of 0.658 as the most sensitive with the highest level of specificity. However, the ROC curve (sensitivity vs. 1-specificity) was basically a 45-degree line running from the intersection of the axes, indicating that H_con_/Q_con_ ratio was no better than chance alone for predicting hamstring injury (area under curve 0.537). Thus, although no useful decision threshold could be identified in this study, for illustrative purposes, in further analysis we have decided to use this value as the “third” cut-off value (apart from values of 0.47 and 0.6) to calculate diagnostic usefulness of isokinetic test in hamstring strain injury diagnosis.

### Sensitivity, specificity

Sensitivity, specificity, accuracy, positive and negative predictive values, positive and negative likelihood ratios and diagnostic odds of the H_con_/Q_con_ ratio for hamstring strain injury prediction, calculated for different cut-off scores are presented in Table 3. Comparison of sensitivities and specificities (calculated among injured football players alone and among non-injured players respectively) is shown in Table 4.

**Table 3:** Pooled estimates of the sensitivity, specificity, positive and negative likelihood ratios and diagnostic odds of the H_con_/Q_con_ ratio for hamstring strain injury prediction, by different cut-off score.

Cut-off score	TP	FP	FN	TN	Sensitivity	Specificity	PPV	NPV	LR+	LR-
					(95% CI)	(95% CI)	(95% CI)	(95% CI)	(95% CI)	(95% CI)
					[%]	[%]	[%]	[%]		
**H**_**con**_**/Q**_**con**_ **<0.47**	2	18	10	310	16.7 (2.1–4.8)	94.5 (91.5–96.7)	10 (1.2–31.7)	96.9 (94.3–98.5)	3.04 (0.79–11.63)	1.67 (0.24–11.43)
**H**_**con**_**/Q**_**con**_ **<0.6**	6	174	6	154	50.0 (21.1–78.9)	46.9 (41.4–52.5)	3.3 (1.2–7.11)	96.2 (92.0–98.6)	0.94 (0.53–1.67)	1.06 (0.59–1.89)
**H**_**con**_**/Q**_**con**_ **<0.658**	11	249	1	79	91.7 (61.5–99.8)	24.1 (19.6–29.1)	4.3 (2.1–7.4)	98.8 (93.2–99.9)	1.21 (1.01–1.45)	0.35 (0.05–2.28)

TP–true positives, FP–false positives, FN–false negatives, TN–true negatives, CI–confidence interval

PPV–positive predictive value

NPV–negative predictive value

LR+–positive likelikood ratio

LR-–negative likelihood ratio

**Table 4:** Comparison of sensitivities and specificities between different cut-offs values (analysed matched-pairs).

Analysed matched-pairs	Sensitivity		Specificity	
	t statistics	P value	t statistics	P value
**<0.47 vs. <0.6**	2.25	0.1336	153.00	<0.0001
**<0.47 vs. <0.658**	6.125	0.0133	229.00	<0.0001
**<0.658 vs. <0.6**	2.25	0.1336	74.01	<0.0001

## Discussion

The current study was aimed to measure the sensitivity and specificity of different cut-off values for the isokinetic H_con_/Q_con_ ratio in order to improve the capacity to retrospectively evaluate the injury risk of hamstrings muscles in professional soccer. The main finding of this study is that application of different cut-off values for H_con_/Q_con_ significantly affects the sensitivity and specificity of isokinetic test used as a tool for muscle injury detection. The use of 0.47 of H_con_/Q_con_ as a discriminate value resulted in significantly lower sensitivity when compared to 0.658 threshold (sensitivity of 16.7% vs. 91.7%, respectively; t = 6.125, p = 0.0133). Calculated values of specificity (when three different cut-off were applied) were also significantly different. Threshold of 0.6 of H_con_/Q_con_ results with significantly lower specificity compared to 0.47 value (specificity of 46.9% vs. 94.5%, respectively; t = 153.0, p<0.0001), and significantly higher specificity when compared to 0.658 (specificity of 46.9% vs. 24.1%, respectively; t = 229.0, p<0.0001). These outcomes suggest that using isokinetic test as a screening tool in a group of male professional football players to predict hamstring injury occurrence within the next 12 months might be significantly biased due to the different cut-off values of H_con_/Q_con_.

According to the Houweling et al. the sensitivity of the test is the most important for the detection of hamstring injury in football players. A test with high sensitivity has low false-negatives (i.e. negative diagnostic test in players that have hamstring injury) and, therefore, the test is more likely to identify any players at risk [13], and probability of being test positive when disease present is higher [39]. A high sensitivity is clearly important where the test is used to identify a serious but treatable disease [40]. Based on the three different cut-offs applied in the statistical analysis in this study, the highest sensitivity (91.7%; 95%CI 61.5%-99.8%) was found for 0.658 H_con_/Q_con_ discriminate value. However, this cut-off threshold results in the lowest specificity (24.1%; 95% CI 19.6%-29.1%). That means that applying 0.658 value results in almost 75% of incorrectly identified players as test positive (false positive), which makes the usefulness of isokinetic evaluation in hamstring injury prediction very questionable. On the other hand, applying 0.47 cut-offs provides a directly opposite calculation: the lowest sensitivity (16.7%; 95%CI 2.1–4.8%) and the highest specificity (94.5%; 95%CI 91.5%-96.7%). Based on these different outcomes it is really difficult to provide clear guidelines for interpreting the results of isokinetic test as a predictive test for hamstring strain injuries. Zvijac et al. calculated the sensitivity and specificity for the hamstrings-to-quadriceps ratio predicting hamstring injury in American football players at 51% (95% CI 41,9%-60,7%) and 52% (95% CI 49.5%-55.4%) indicating that the hamstrings-to-quadriceps ratio was not a useful predictor of injury (calculation used the mean ± SD ratio for injured legs, 0.656 ± 0.133) [41]. Conversely, although Dauty et al. [9] reported only 3.1% for sensitivity and 98.2% for specificity of isokinetic H_con_/Q_con_ in hamstring strain injury prediction for H_con_/Q_con_<0.47, they found that according to injury severity H_con_/Q_con_ ratio was useful in prediction of moderate and major injuries. However, is has to be remembered that any comparison of these results might be difficult and biased due to the different populations tested, various injury definitions, and different cut-off thresholds which influenced the numbers in the 2 x 2 contingency table for compared variables: predicted injured or uninjured with actual injured or uninjured.

Sensitivity and specificity values may provide useful information, but they have several shortcomings; these values cannot be used to quantify the shift in probability of the condition given a certain test result [42]. It is also reported that sensitivity and specificity are useful when test results are going to be compared with the “gold standard”. However, in the clinic, also in isokinetic assessment, it is difficult to establish the “gold standard”.

Bahr has underlined lately, that more appropriate statistical measures than sensitivity, specificity, positive and negative predictive values and odds ratios, should be used to describe the predictive ability of a screening test, such as likelihood ratio or receiver operating characteristic curve analyses [43]. Likelihood ratios are considered the best statistics for summarizing the usefulness of a diagnostic test; sensitivity and specificity work in the opposite direction of clinical decision-making, while predictive values are highly dependent on the prevalence of the condition of interest in the sample [42]. Since likelihood ratios are ratios of probabilities, they can be treated as risk ratios for the purposes of calculating confidence intervals. However, likelihood ratio (negative and positive) still relies on 2 x 2 table with the usage of discriminative values. It is therefore necessary to understand that obviously, the accuracy of likelihood ratios depends on the quality of the studies that generated the numbers (sensitivity and specificity). Moreover, as the likelihoods are calculated from sensitivity and specificity, like these parameters they too may be affected by validation, precision, severity of injury/disease or variation from study to study [44].

Therefore, ROC analysis seems to be the most appropriate statistical method to establish the best cut-off in injury related studies, because it compares test accuracy over different cut-off scores and, consequently, eliminates the probability of it being selected arbitrarily [13]. However, ROC analysis provides one–“the best” value of cut-offs, determining the likelihood of injury occurrence. This discriminative threshold is used to classify whether an athlete is at risk of injury or not. But football players tend to be asymmetrical in lower leg strength mainly attributed to preferred sidedness in executing most of the unilateral football skills [45]. In the study of Rahnama et al., 28 of 41 players (68%) were found to have at least one musculoskeletal abnormality which consisted of a contralateral strength imbalance of greater than 10% [46]. This propensity is also reported by Fousekis et al. 39 of 100 football players (39%) of who underwent a preseason evaluation (and who were free of injury for at least 6 months prior to testing) had isokinetic strength imbalances in the knee joint ≥15%; moreover, dispersion of the standard deviation for conventional H_con_/Q_con_ ratio ranged from 0.10 to 0.80, and additionally 95% of confidence interval ranged from 0.26 to 0.86 [45]. Thus, it might not be an appropriate approach to use one “normative” value as a cut-off (e.g. 0.47 or 0.60, or any other calculated from ROC analysis) to qualify a football player to a group with higher risk of knee injury. In our opinion, assigning some players (who had H_con_/Q_con_ ratio<normative threshold in pre-screening tests at the beginning of the observational period) to the group “at risk of injury”, and drawing conclusions on the relation between isokinetic strength and hamstring injury might be biased due to the natural asymmetry observed among football players. The probability of muscle injury risk determined based on only one cut-off value might not be sufficient when applied to the whole group.

That is why we would like to suggest that for establishing the “normative” values for H_con_/Q_con_ ratio we need to think of “H_con_/Q_con_ range” that might be helpful in the implementation of the existing football-specific asymmetry, and intra-player dispersion of H_con_/Q_con_ value in the injury risk assessment. In our opinion the injury risk analysis performed with isokinetic tests should not based on a dichotomous approach (equal to and higher or lower than the cut-off value) but based on the range that might provide more suitable results. Extending the threshold in the current study by about 10% (from 0.6 –to 0.658) resulted in five more cases of true positive (increasing sensitivity from 50% to 91.7%, Table 3). What is more, the specificity of the isokinetic test also rises. That might provide slight evidence that it might be more appropriate to classify a player to the injury risk group based on the extended range. In a large number of studies performed in a population of football players conclusions were drawn based on the 0.47 value of H_con_/Q_con_ ratio [7,9,10]. However, their authors seem to be unaware of the origins of the 0.47 cut-off value for H_con_/Q_con_, and hence of its inappropriacy. Moreover, several cross-sectional studies performed on a large sample of professional soccer players reveal different characteristics for this study population with H_con_/Q_con_ value for non-injured footballers or for non-injured leg often higher than 0.55 (Table 1). Concentric H/Q ratio in uninjured players was lower in our group when comparing to the Portugal [4] and French [9] professional players. Mean value in our study group was at 0.6±01, whereas soccer player from Portugal achieved 0.61±0.1 and 0.62±0.1 for left and right leg, respectively [4]. French footballers presented even higher values. They achieved 0.66±0.1 for uninjured and similar 0.66±0.11 for injured leg [9]. It was considerably higher compared to our population, in which injured players presented H/Q value at 0.58±0.09 for injured leg. English Premier League soccer players presented also higher value of H/Q concentric ratio for injured players when compared to our cohort; it was 0.60±0.09 for injured and 0.62±0.12 for non-injured dominant leg [28]. These results for injured leg are much higher than the 0.47 suggested by Croisier et al., who believed that values lower than 0.47 indicated a higher injury and re-injury risk [7,21].

Therefore, we would like to propose a model of assigning players to either predicted uninjured or predicted injured group based on preseason isokinetic tests using two types of variables: assignment to the predicted uninjured group when the player’s results are within the “normal” range of H_con_/Q_con_ ratio (e.g. H_con_/Q_con_ from 0.6 to 0.66), and assignment to predicted injured group when the H_con_/Q_con_ ratio is lower or higher than the extreme values. However, due to the fact that H_con_/Q_con_ ratio is different depending on sex, age, position on the field [27] and level of competition [4], it might be challenging to define the “normal” range of value. Additionally, further studies should focus on establishing a more suitable range for professional football players. Nevertheless, we believe that future studies might provide less biased results than today, when injury prediction using isokinetic test is based on “the one and only correct” H_con_/Q_con_ value.

Within the current manuscript data was analysed from the Polish Premier League, that have a winter break, and in which the isokinetic tests are performed usually twice a year: in the January and June/July (directly before the preseason’s conditioning periods). In England [28,47] and Portugal [4] the isokinetic tests are performed during the preseason, usually in July, whereas in France soccer players are assessed at the beginning of the soccer season [9]. In Qatar Stars League the isokinetic evaluation is performed from May to September [12]. In some European leagues official football season starts in mid-July and it ends around May/June, and there is no winter break during the season [4,9,28]. Similarly, in Qatar there is no break, however football season begins usually mid-September and it continues through mid/end of April [12]. In Poland the season starts much earlier (in mid-July) and it ends around May/June with the winter break from mid-December to mid-February. Since there are season structure differences between Poland (likewise few other leagues in Central Europe, e.g. Bulgaria, Czech Republic, Slovakia) and other European countries like England, Italy, France, Spain or Qatar, the results from our study should be applied to the European leagues with caution. Especially when comparing our data with studies done in leagues without winter break (i.e. English Premier League, Serie A, Ligue 1 and La Liga), in where the strength tests are usually done after the summer off-season.

It seems very difficult to provide clear-cut recommendations for the association between isokinetic tests and sustained injuries, since the literature is still not too homogeneous. When it comes to the methodological details it appears, that there are some seemingly minor differences that can produce markedly divergent outcomes (the differences may include e.g. the proper population, the cut-off value, or different structure of the football season). Therefore, more studies are needed to confirm the results in other European competitive national leagues.

## Conclusions

The use of different cut-off values for H_con_/Q_con_ significantly affects the sensitivity and specificity of isokinetic testing. The interpretation of usefulness of isokinetic test as a screening tool in a group of male professional football players to predict hamstring injury occurrence within the next 12 months might be therefore significantly biased due to the different threshold values of H_con_/Q_con_. Using one “normative” value as a cut-off (e.g. 0.47 or 0.60, or any other calculated from ROC analysis) to qualify a football player (or not) to the group with a higher risk of knee injury might result in biased outcomes due to the natural strength asymmetry that is observed within the group of football players. The authors studied retrospectively a Centre Europe national league (with winter break) so the results may be extended across other European competitive leagues.

## Limitation of the study

The main limitation of this study is the retrospective study design, and these non-experimental studies provide evidence only of an association between the factor and risk of injury [48]. We also had no information regarding exposure time or other subject-related risk factor. Another issue, which might influence the results, is associated with specificity of treatment of professional football players. Even though there is usually a medical cooperation agreement between the club and the medical clinic, the player can seek and obtain other methods of treatment or consultation; and the main physician may not even know about treatment provided by other clinics/specialists. During the analysed period a low number of hamstring strain injuries occurred, and results from this study should be verified on a bigger sample with longer hamstring injury observation to identify significant interrelations. Regarding hamstring strain injury, it also has to be emphasized that we have analysed only injuries confirmed in USG evaluation. It is possible that some muscle strain might have been missed. Since we have no data regarding the exposure time, we have not calculated the injuries as number injuries / 1000h of training/competitions of the team (each year or average during the data collecting period). Such a calculation would strongly improve the descriptive of the sample.

## Supporting information

S1 File Statement of the Bioethical Committee. Polish translation from the Bioethical Committee regarding the ethical issues.
(PDF)
