# Supplementary material for: Discussion about different cut-off values of conventional hamstring-to-quadriceps ratio used in hamstring injury prediction among professional male football players
Source: PLoS One. 2017 Dec 7;12(12):e0188974. doi: 10.1371/journal.pone.0188974 (PMC5720729; doi:10.1371/journal.pone.0188974)
Supplement: S1 File — Polish translation from the Bioethical Committee regarding the ethical issues. (PDF) [file pone.0188974.s001.pdf]

Małgorzata Zgrych
Certified translator/interpreter of the English language
ul. Niemcewicza 16g/10, 71-520 Szczecin
Phone: 501 312 004
Email: malgorzata.zgrych@dobretlumaczenia.eu

CERTIFIED TRANSLATION FROM POLISH

[the document consist of one page]

[in the upper left-hand corner, the official logo of the Poznań University of Medical Sciences]

THE KAROL MARCINKOWSKI POZNAŃ UNIVERSITY OF MEDICAL SCIENCES-/-

BIOETHICS COMMITTEE OF THE KAROL MARCINKOWSKI POZNAŃ UNIVERSITY OF MEDICAL SCIENCES-/-

Collegium Maius	Phone: (+48 61) 854 62 51-/-
ul. Fredry 10	Fax: (+48 61) 854 61 07-/-
61-701 Poznań	www.bioetyka.ump.edu.pl-/-

KB no. 1238/16-/-

STATEMENT-/-

The scientific research entitled "***A retrospective analysis of interdependencies between the functional and biomechanical evaluation of athletes and lower limb injuries in a group of professional footballers***"-/-

conducted by **Ms Monika Grygorowicz, PhD in Physical Culture**-/-

does not have the characteristics of a medical experiment and, according to the Polish law and the Good Clinical Practice, does not require review by the Bioethics Committee.-/-

Poznań, **1 December 2016**-/-

Committee Chair-/-
[handwritten signature]

Professor Paweł Chęciński, MD, PhD

XXXXXXXXXXXXXXXXXXXXXXXXXXXXXXXXXXXXXXXXXXXXXXXXXX

I, Małgorzata Zgrych, sworn translator of the English language, entered into the list of sworn translators kept by the Polish Minister of Justice under entry no. TP/45/14, hereby attest that the above is a true and complete translation of the scanned original document in Polish presented to me.

Register 22/2017

Szczecin, 1 March 2017

Małgorzata Zgrych

MAŁGORZATA ZGRYCH TŁUMACZ PRZYSIĘGŁY JĘZYKA ANGIELSKIEGO NR TP/45/14
